# Supplementary material for: Current khat (Catha edulis F.) use among Ethiopian women and its association with anemia and underweight: A cross-sectional analysis from Ethiopian Demographic and Health Survey
Source: PLoS One. 2024 May 31;19(5):e0297831. doi: 10.1371/journal.pone.0297831 (PMC11142676; doi:10.1371/journal.pone.0297831)
Supplement: S1 Table — (DOCX) [file pone.0297831.s001.docx]

S1 Table. Bivariate and multivariate analysis showing association between current khat chewing status and malnutrition using pearson’s chi-square

|  |  | | Total | Currently Chewing | | | Chi-square | p-value |
| --- | --- | --- | --- | --- | --- | --- | --- | --- |
|  |  | |  | No | | Yes (%) | 5.4972 | 0.139 |
| Anemia level | Severe | | 144 | 133 | | 11 (7.6) |  |  |
|  | Moderate | | 989 | 890 | | 99 (10) |  |  |
|  | Mild | | 2,796 | 2,575 | | 221 (7.9) |  |  |
|  | Not anemic | | 10,560 | 9,724 | | 1192 (11.3) |  |  |
| BMI | <17 | | 1,215 | 1,149 | | 66 (5.4) | 40.66 | < 0.001 |
|  | 17-18.4 | | 2,137 | 1,963 | | 174 (8.1) |  |  |
|  | 18.5-24.9 | | 8,534 | 7,862 | | 672 (7.9) |  |  |
|  | 25 – 29.9 | | 1,194 | 1,071 | | 123 (10.3) |  |  |
|  | >=30 | | 354 | 302 | | 52 (14.7) |  |  |
| Anemia | | Non-Anemic | 11,395.84 | 10,203.89 | 1,191.94 | | 0.9973 | 0.318 |
|  | | Anemic | 3,526.999 | 3,115.172 | 411.827 | |  |  |
| BMI | | Not-Underweight | 10,581.03 | 9,517.895 | 1,063.13 | | 5.2114 | 0.022 |
|  | | Underweight | 3,063.088 | 2,720.276 | 342.812 | |  |  |
