# Supplementary material for: Current khat (Catha edulis F.) use among Ethiopian women and its association with anemia and underweight: A cross-sectional analysis from Ethiopian Demographic and Health Survey
Source: PLoS One. 2024 May 31;19(5):e0297831. doi: 10.1371/journal.pone.0297831 (PMC11142676; doi:10.1371/journal.pone.0297831)
Supplement: Attachment — Submitted filename: Review report on Prevalence and correlates of khat.docx [file pone.0297831.s002.docx]

Review report on Prevalence and correlates of khat (Catha edulis F.) use, anemia, and underweight in Ethiopian women: A cross-sectional analysis from Ethiopian Demographic and Health Survey

| **No case** | **Part** | **Issues** | **Recommendation** |
| --- | --- | --- | --- |
| 1 | From title to conclusion |  |  |
|  |  | The title, the abstract, the objective, and the result are not in line | Revised from abstract to conclusion part based on the data that you have |
|  |  | Lack of concentration or focus according to the topics |  |
|  |  | Poor language use | Needs language editor |
|  |  | Do you think your study is different from Prevalence and determinants of chewing khat among women in Ethiopia: data from Ethiopian Demographic and Health Survey 2016 | You have to focus on the issues that is new compared with the previous study. |
|  |  | The same study with [Yimenu Yitayih](https://bmcpsychiatry.biomedcentral.com/articles/10.1186/s12888-021-03136-y#auth-Yimenu-Yitayih-Aff1) &  [Jim van Os](https://bmcpsychiatry.biomedcentral.com/articles/10.1186/s12888-021-03136-y#auth-Jim-Os-Aff2-Aff3-Aff4)  study above but your report is different from the previous one. Why? | Go to your data and look again the way of cleaning and inclusion so on… |
| 2 | Abstract | Not structure | structure |
|  |  | Lacking a study population, sampling, and data collection |  |
|  |  | Lacking the absolute number of participants, the result of prevalence and its CI |  |
|  |  | The conclusion without recommendation |  |
|  |  | No keyword |  |
| 3 | Introduction | Line 60-61: What is STEEP |  |
|  |  | Line 85-88: Long sentence |  |
| 4 | Method | Poor structure | =>Study design and sampling; =>study variables and Measurements  =>Data analysis |
|  |  | 115-126: Unclear information, Why did you report other studies here?  Is it a case-control study? |  |
|  |  | Line 131 and 139: Measurements |  |
| 5 | Result | Line 155-161:Poor summary of Socio-demographic profile | Write the Mean age and rewrite the summary |
|  |  | Line 155: Prevalence without Confidence internal. It is not part of the Socio-demographic profile |  |
|  |  | 158: adhered to the Orthodox Christian faith. What do you mean? |  |
|  |  | Socio-demographic characteristics of current khat chewers | Line: 170-185  Rewrite it with clear statements |
|  |  | No report on the prevalence of malnutrition |  |
|  |  | Line 212: Association between khat chewing frequency and malnutrition. Why do we use only age as a predictor variable? |  |
| 6 | Discussion | It is not concise because of the title objective and result | Set inline among topics: the title, objective, result, and discussion. For example:  The title "….. khat (Catha edulis F.) use, anemia, and underweight in Ethiopian women:…" but you did not report much on the Prevalence and determinants of chewing khat among women. The report on anemia, and underweight is poor and not stated well enough to make it different from the previous study |
| 7 | Conclusion and recommendation | Line 431-432: I did not see it in the regression, but you reported it as an associated factor. | Focus on results |
|  |  | Line 434-435 : women who chewed khat were found to be more susceptible to being underweight than non-chewers. How did measure susceptibility? Where is it in the results? |  |
